# Prevalence and perception of substance abuse and associated economic indicators and mental health disorders in a large cohort of Kenyan students: towards integrated public health approach and clinical management

**DOI:** 10.1186/s12888-022-03817-2

**Published:** 2022-03-17

**Authors:** Victoria N. Mutiso, David M. Ndetei, Esther N.Muia, Christine Musyimi, Tom L. Osborn, Rita Kasike, Lydia Onsinyo, Jane Mbijjiwe, Pamela Karambu, Andre Sounders, John R. Weisz, Monica H. Swahn, Daniel Mamah

**Affiliations:** 1 Africa Mental Health Research and Training Foundation, Nairobi, Kenya; 2 World Psychiatric Association Collaborating Centre for Research and Training, Nairobi, Kenya; 3 Department of Psychiatry, University of Nairobi, Kenya and Africa Mental Health Research and Training Foundation, Mawensi Road, Off Elgon road, Mawensi Garden, P.O. Box 48423-00100, Nairobi, Kenya; 4 Department of Public and Community Health, Machakos University, Machakos, Kenya; 5 Department of Psychology, Harvard University, Cambridge, MA USA; 6 Shamiri Institute, Allston, MA USA; 7 Shamiri Institute, Nairobi, Kenya; 8 Department of Child Psychiatry, Turku University Hospital, Turku, Finland; 9 Department of Population Health Sciences, Georgia State University, Atlanta, USA; 10 Department of Psychiatry, Washington University Medical School, St. Louis, MO USA

**Keywords:** Student, Substance use, Economic indicators, Mental health disorders, Interventions, Kenya

## Abstract

**Background:**

The earlier younger people begin to use drugs, the more vulnerable they become to both their short term and long-term harmful effects. The overall aim of this study is to determine the prevalence of alcohol and drug abuse, the socio-demographic characteristic, perception of abuse and associated economic indicators and mental disorders and how they inform potential intervention in a cohort of Kenyan students.

**Methods:**

This was a cross-sectional study on a total of 9742 high school, college and university students. We used tools to document socio-demographic characteristics, economic indicators, drug and alcohol use and related perceptions and Diagnostic and Statistical Manual of Mental Disorders, Fourth Edition (DSM-IV) related psychiatric disorders. Basic descriptive statistics (means and standard deviations for numerical variables and frequencies for nominal and ordinal variables) were done. Logistic regression models were used to assess the association and odds ratios between the use of a given substance and the use of the other substances, as well as associations with the various available socio-demographic factors and economic indicators. Chi-squared tests were used in socio-economic characteristics disaggregated by current alcohol use.

**Results:**

The mean age was 21.4 ± 2.4; median = 21.3 (range 15–43) years. We found a wide range of different drugs of abuse. Alcohol abuse was the commonest and inhalants were the least, with different perceptions.Both alcohol and drug abuse were associated with various economic indicators and various mental disorders.

**Conclusion:**

This study has established for the first time in Kenya the multifaceted associations and predictors of alcohol and drug abuse in a cross-sectional student population ranging from high school to college and university levels. In the process, the study contributes to global data on the subject. These associations call for an integrated and multifaceted approach in addressing alcohol and substance abuse. This approach should take into account various associations and predictors as part of holistic approach in both public awareness and clinical interventions.

**Supplementary Information:**

The online version contains supplementary material available at 10.1186/s12888-022-03817-2.

## Background

Substance use among youth is harmful and has been associated with increased risk for a number of well-established consequences including: impaired peer relationships, mental illness, increased risk for suicide, high-risk sexual behavior, HIV/AIDS, disrupted learning, truancy, school drop-out and poverty [1, 2]. However, these studies were on co-morbidity rather than directional association. The earlier younger people begin to use drugs, the more vulnerable they become to both short term and long-term harmful effects [3, 4]. Alcohol abuse contributes the highest burden of substance use disorders worldwide [5, 6]. Annually, 320,000 young people aged 15–29 years die from alcohol-related causes, resulting in 9% of all deaths in that age group globally [6].

The National Institute of Drug Abuse [7, 8] reported several factors that can increase the risk of initiating or continuing substance use. These factors include: socioeconomic status, quality of parenting, peer group influence, and biological/inherent predisposition toward drug addiction. Fixed markers for increased vulnerability include sex, biological indicators, income, family substance history, parent psychopathology, parental marital status and income/social economic status [8]. Key risk periods for drug abuse occur during major transitions in young people’s lives during which they face additional social, psychological, and educational challenges, complicated by exposure to greater availability of drugs, drug abusers, and social engagements involving drugs [7]. On abuse of psychoactive substances in general globally, (including heroin, crack, cannabis, prescription drugs among others) there has been a rise among the youth [9].

Studies show that substance use has increased among African youth [10], posing serious social and public health problems similar to those in most Western societies [11, 12]. Among the estimated 269 million users of any drug, some 35.6 millionare estimated to suffer from substanceuse disorders, meaning that their drug use is so harmful that they may experience drug dependence and/or require treatment [13].

In Ethiopia, the commonly used substances were found to be alcohol, cigarettes, khat and cannabis which frequently lead to addiction [14]. In Zambia the lifetime prevalence of cigarette use has been reported at 20.5% among 15 year old adolescents, and even higher (37.2%) among males younger than 12 years old [15]. Compared to Kenya, where it is reported to be at 42.8% in Kenyan college students; at 32.2% prevalence of lifetime smoking in Nairobi, the capital City of Kenya [8]. In Kenya, lifetime prevalence rate of any substance use was found to be 69.8% among college students [16]. According to a national survey conducted every 5 years by theNational Authority for the Campaign Against Alcohol and Drug Abuse (NACADA), lifetime usage of alcohol, tobacco, and Khat among 15 to 65 year olds in Kenya stood at 18.3, 12.8, and 7.2% respectively; use of the same substances by 15–24 year olds was18.6, 5.2, 6.7% respectively and 2.4% for bhang as at 2017 [17]. This data was a sample of 3,136 households distributed across all the regions in Kenya [17].

Some of the known risk factors (such as; low self-esteem, psychopathology, poor relationship with parents, among others) that prompt substance use among the youth have been stated to be universally applicable [16]. A study conducted in Kenya also concluded that some factors such as; the influence of the media; levels of education (low); culture – where some youth perceive usage of substances as appealing and have some form of preeminence and therefore those who do not use them are esteemed in low regard; and the low cost of drugs making them easily accessible [18].

It is of concern that tobacco is a second component of abuse given that it is a highly addictive substance likely to continue into later age, with all the known medical complications. Further, unlike alcohol, which is easily affordable and widely available, cigarettes are much more expensive. Their availability is highly restricted by law in that retailing is limited to a pack of 20 rather than single sticks of cigarettes. It is possible that even if the retail outlets only allowed packet of 20, youth or their of age agents could still pool resources for one of them to buy a packet of 20 and then share and in the process circumvent the legal requirements not to sell single cigarette. Cannabis is widely available. Its trafficking, though prohibited by law, still occurs. In the Kenyan context the psycho-stimulant khat which acts like amphetamines is widely available and lawful. Growing and marketing of khat is allowed by the Government. Of concern are the sedatives which users of psycho-stimulants use to bring them down. Therefore, the similarity in the use of khat/amphetamine and sedatives is not surprising. But unlike khat they are prescription drugs. Obviously they are being obtained through fraudulent prescriptions or unscrupulous pharmacies. Hallucinogens, Cocaine and Opioids, though still at very low prevalence possibly because of high costs, are used by Kenyan youth. Inhalants from glue and petrol and which cost nothing to obtain, are associated with homeless street children, usually in urban areas.

A major shortcoming of most studies on substance use in Africa and Kenya in particular, have been limited mainly to only epidemiological patterns and have tended to be regional rather than national apart from NACADA studies. More research is needed to improve our understanding of the nuances that underline substance use and have the potential to inform development of interventions.

The overall objectives of this study are to go beyond the prevalence of substance abuse in schools and colleges to include universities, to examine for the first time in Kenya the students' perceptions on alcohol and substance abuse and to go beyond socio-demographic factors to include quantifiable wealth index as an economic indicator for the first time in Kenya. All of these provide more inclusive context appropriate evidence that can inform interventions in the Kenyan context from different but interactive perspectives.

### The specific aims of this study are:

1. To determine the prevalence of different types of drug use in High schools, College and University students in one combined study.
2. To understand what the students themselves thought about substance and alcohol use.
3. To assess associations among socio-demographic and economic factors and current substance useand psychiatric disorders.
4. To determine the independent predictors of substance and alcohol use
5. To suggest possible interventions.

The above general objectives and specific aims will be achieved by answering the following questions:

1. what are the comparative prevalencesof substance abuse in high school, college and university students?
2. What are the studentsperceptions of alcohol and substance abuse?
3. What are the associations between socio-economic factors and substance and alcohol abuse?
4. What are the independent predictors of alcohol and substance abuse?
5. Is there potential for multifaceted informed approach to intervention?

## Methods

### Recruitment

Participants were recruited from University, community and mid-level colleges in four out of the 47 counties in Kenya,thatis:Nairobi, Machakos, Kitui, and Makueni. These counties were conveniently chosen because of other community based programs that the supporting Institution, Africa Mental Health Research and Training Foundation (AMHRTF), was undertaking in these areas. Inclusion criteria were informed voluntaryconsent for those aged 18 + and parental consent and child assent for those aged < 18.A total of 9,742 subjects participated in the study with the majority (87%) being recruited from university and college campuses. The high school students were recruited from communities because at the time of the study the schools were closed because of an ongoing national strike by the teachers. We went through the community leadership to inform the administrators and parents of this study with request to inform the students to meet at a specified community hall. We obtained informed consent from the parents and informed assent from those under 18 years. All the instruments were researcher administered.

### Tools:

These tools were selected to enable us answer our research questions in order to achieve our general objectives and specific aims. They were part of a bigger study to determine predictors of early psychosis in Kenya.

1. Socio-demographic questionnaire designed by the researchers -These questions were selected in order to determine the correlation of various factors and substance use among school, college and university students.
2. The Wealth Index Questionnaire -The wealth index used is based on the World Bank Recommendation for Low and Middle Income Countries (LMIC) [19] and has been adopted by the Kenya Government for use in Kenya. The developers of this instrument borrowed heavily from tools that have been used in several contexts in high income countries which in their own did not provide any psychometric properties. The adopted version for Kenya did not also provide psychometric properties nor did it have any gold standards. This tool has questions regarding household items, type of housing, water source, toilet type and source of energy for cooking. These items are used to estimate socio-economic status by creation of wealth index, grouped into five quintiles with quartile 1 representing the lowest level of wealth and 5 the highest level.
3. TheAlcohol, Smoking, and Substance Involvement Screening Test (ASSIST) [20]. This instrument was developed by World Health Organization (WHO) specifically for use in low and middle income countries and piloted in several countries before the WHO recommended it for use in low and middle income countries. The ASSIST collects information on, and determines levels of risk from, the use of tobacco products, alcohol, cannabis, amphetamine-type stimulants, cocaine, sedatives and sleeping pills, hallucinogens, opioids, and “other” drugs. This tool takes about five minutes to complete and has been validated for use in LMICs [21]. Each substance is the object of eight questions to establish its lifetime use (Question 1); frequency of use in the past three months (Question 2); frequency of experiencing a strong desire or urge to use each substance in the last 3 months (Question 3); frequency of health, social, legal, or financial problems related to substance use in the last 3 months (Question 4); frequency with which use of each substance has interfered with roles or responsibilities in the past 3 months (Question 5); whether anyone has ever expressed concern about the respondent’s use of each substance, and how recently that occurred (Question 6); whether the respondent has ever tried to cut down or stop the use of a substance, and failed in that attempt, and how recently that occurred (Question 7); and whether the respondent has ever injected a substance, and how recently that occurred (Question 8). Responses to questions 2 through 7 of the ASSIST generate a score indicating the level of risk associated with the respondent’s use of each category of substance. Risk is classified as: low risk (0 to 10 for alcohol, and 0 to 3 for all other substances); moderate risk (11 to 26 for alcohol, and 4 to 26 for the other substances); and high risk (27 and above) [20].
4. The Psychiatric Diagnostic Screening Questionnaire (PDSQ)—We picked PDSQ tool. For the purpose of this report, we focused on questions 1 and 2. Subscales on alcohol and substance abuse/dependence to assess for drug and substance dependence and also the PDSQ psychiatric disorders. Although PDSQ was originally designed for clinical population, it has also been used for non-clinical population. The PDSQ is a psychometrically strong self-report scale [22, 23], used for screening of DSM-IV axis I disorders, with a high negative predictive value and good sensitivity in its subscales [23]. It determines dependence based on individual perceptions on alcohol and substance use and makes DSM-IV related psychiatric disorders.

### Statistical analysis

Data was coded, checked, cleaned and exported to Statistical Package for the Social Sciences (SPSS) for analysis. Basic descriptive statistics (means and standard deviations for numerical variables and frequencies for nominal and ordinal variables) were done. For tobacco, alcohol, sedatives, Khat, cannabis, and cocaine, the most prevalent lifetime substance use, logistic regression models were used to assess the association and odds ratios between the use of a given substance and the use of the other substances, as well as associations with the various available socio-demographic factors and economic indicators. Chi-squared tests were used in Socio-economic characteristics disaggregated by Current alcohol use (In supplementary Table 2).

## Results

### Socio-demographic characteristics

Table 1 summarizes the socio-demographic characteristics (frequencies and percentages) of the respondents.Participants’ mean age was 21.4 (± 2.4) years. More than half of the participants were male, majority were single (93.4%), university students (68.6%), 1st or 2nd born (56.9%), protestant and catholic were the commonest religions, and the wealth index was similar across all students.

**Table 1:** Socio-demographic characteristics of respondents (*N* = 9742)

Variable	Category	Frequency (*N* = 9742)	Percentage (%)
Sex	Male	5173	53.5
	Female	4500	46.5
	*Missing*	*69*	
Age	Mean ± SD; Median; Range	21.4 ± 2.4; 21.3; 15–43	
Marital status	Married	607	6.3
	Single	9057	93.4
	Others	38	0.4
	*Missing*	*40*	
Religion	Protestant	5512	57.1
	Catholic	3359	34.8
	Muslim	410	4.2
	Other	368	3.8
	*Missing*	*93*	
Birth order	1–2	5539	56.9
	3–5	3271	33.6
	6 +	920	9.5
	*Missing*	*12*	
Level of Education	High School	1506	15.5
	College	1534	15.8
	University	6648	68.6
	*Missing*	*54*	
Wealth Index	Quintile 1	1944	20.1
	Quintile 2	1944	20.1
	Quintile 3	1902	19.7
	Quintile 4	1951	20.2
	Quintile 5	1928	19.9
	*Missing*	*73*	

### Substance use over time

**Fig. 1:**
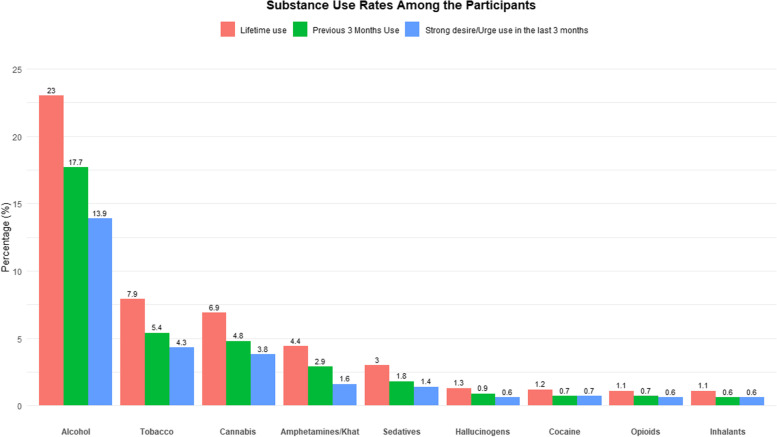
Drug and substance Use among the respondents. Note: this figure gives proportion of assist instrument (Qs 1–3)

Figure 1 summarizes the lifetime use, use in last 3 months and current strong desire from the WHO ASSIST Tool for the various substances. These are arranged in a descending order of frequency. Alcohol was the most used substance while inhalants were the least used.

### Current index perception of students use of alcohol and substance abuse

**Fig. 2:**
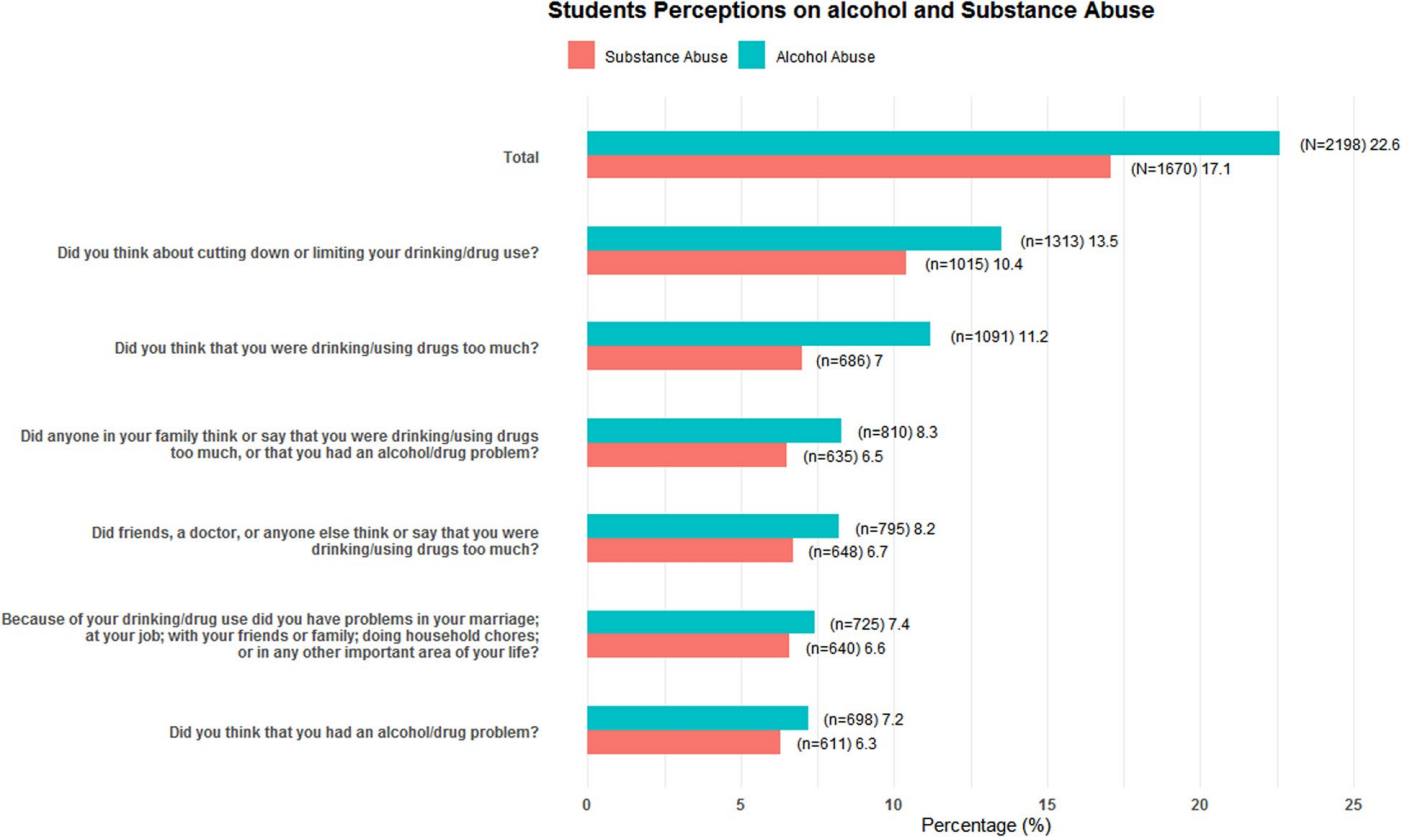
Student perceptions on alcohol and substance abuse. Note: this figure gives proportional comparisons of perceptions of substance use and alcohol abuse. Substance abuse is a continuous variable which represents all other substances a part from alcohol. (More details in supplementary table 1.)

Figure 2 summarizes the perceptions of alcohol and substance abuse based on the PDSQ scores on questions on alcohol/substance abuse and compares the perception on alcoholalongsidesubstance use for each of the different questions but re-arranged in a descending order. The legend for Fig. 2: *N* = total of either alcohol or substance use; *n* = number for each perception followed by frequency i.e. (n or N)/9742 × 100. There were different perceptions for alcohol and substance abuse. The different perceptions varied in frequency.The question on whether they thought they had any problem with alcohol or substance use was the least endorsed (more details in a table are summarized in supplementary table 1).

### Substance abuse and associated socio-demographic factors

#### *Independent predictors of Lifetime substance use* 

We summarize these in a narrative:

- For tobacco use in the lifetime multivariate model, male sex(A.O.R 3.89 (CI 3.23–4.69)), older age (A.O.R 1.11 (CI 1.08–1.15)), not being married /not single, being 6^th^ born and above in the family (A.O.R 0.65 (CI 0.47–0.88)), and wealth index Quintile 2 (A.O.R 1.83(CI 1.45–2.32)) and Quintile 4 (A.O.R 0.69 (CI 0.53–0.91)) were significant predictors of tobacco.
- For Alcohol use in the lifetime multivariate model, male sex(A.O.R 1.92 (CI 1.73–2.12)), age (A.O.R 1.09 (CI 1.07–1.12)), not being married /not single, being a catholic(A.O.R 1.23 (CI 1.11–1.37)), being a Muslim(A.O.R 0.39 (CI 0.28–0.53)), higher siblings birth-order (3–6 +), high school students (A.O.R 0.65 (CI 0.56–0.77)),and wealth index Quintile 2 (A.O.R 1.80(CI 1.55–2.09)) and Quintile 4 (A.O.R 0.60 (CI 0.50–0.71)) were significant predictors of alcohol use.
- Cannabis use in the lifetime multivariate model was significantly predicted by male sex(A.O.R 3.21 (CI 2.66–3.90)), older age(A.O.R 1.92 (CI 1.73–2.12)), being single (A.O.R 1.75 (CI 1.15–2.78)), not being married /not single, higher siblings birth-order (3–6 +) and wealth index (Quintile 1 to Quintile 4).
- Sedatives use in the lifetime multivariate model was significantly predicted with age (A.O.R 1.10 (CI 1.04–1.15)) and wealth index Quintile 2 (A.O.R 1.49(CI 1.04–2.17)).
- Khat/amphetamine use in the lifetime multivariate model was significantly predicted with male sex (A.O.R 2.65 (CI 2.11–3.35)),age (A.O.R 1.07 (CI 1.03–1.12)),being a catholic (A.O.R 1.25 (CI 1.01–1.54)) and wealth index Quintile 2 (A.O.R 1.49(CI 1.10–2.03)).

#### Socio-demographic factors associated with current use and independent socio-demographic predictors of current substance use (previous 3 months).

These are summarized in Tables 2 and 3 respectively, with the significant associations (*p* < 0.05) highlighted for quick reference.

**Table 2:** Socio-demographic factors associated with current substance use^a^

Variable	Category	Tobacco Current	Alcohol Current	Cannabis Current	Sedatives Current	Khat/Amphetamines Current
		**O.R (95% C.I)**	**O.R (95% C.I)**	**O.R (95% C.I)**	**O.R (95% C.I)**	**O.R (95% C.I)**
Sex	Male	3.72	1.77	2.74	1.02	2.71
		(2.99–4.67)***	(1.59–1.98)***	(2.21–3.41)***	(0.75–1.39)	(2.07–3.58)***
	Female	Ref	Ref	Ref	Ref	Ref
Age	Mean(SD)	1.08	1.08	1.05	1.08	1.11
		(1.04–1.11)***	(1.06–1.11)***	(1.01–1.09)*	(1.01–1.14)*	(1.06–1.16)***
Marital status	Married	Ref	Ref	Ref	Ref	Ref
	Single	1.37	1.13	1.36	1.50	0.76
		(0.91–2.15)	(0.90–1.42)	(0.89–2.19)	(0.76–3.56)	(0.50–1.22)
	Others	6.45	3.05	3.13	2.21	2.98
		(2.53–15.1)***	(1.49–6.04)**	(0.88–8.78)*	(0.12–12.9)	(0.84–8.33)
Religion	Protestant	Ref	Ref	Ref	Ref	Ref
	Catholic	1.23	1.27	1.12	1.04	1.33
		(1.02–1.49)*	(1.14–1.42)***	(0.91–1.37)	(0.75–1.45)	(1.04–1.71)*
	Muslim	1.25	0.61	1.31	1.48	1.66
		(0.79–1.88)	(0.43–0.83)**	(0.82–1.99)	(0.72–2.72)	(0.96–2.70)
	Others	0.84	0.84	1.45	0.81	0.95
		(0.47–1.38)	(0.62–1.13)	(0.91–2.21)	(0.28–1.80)	(0.45–1.78)
Birth order	1–2	Ref	Ref	Ref	Ref	Ref
	3–5	0.82	0.87	0.77	1.06	1.04
		(0.67–0.99)*	(0.78–0.98)*	(0.62–0.95)*	(0.76–1.46)	(0.80–1.34)
	6 +	0.64	0.65	0.55	0.82	1.35
		(0.44–0.90)*	(0.53–0.80)***	(0.36–0.80)**	(0.44–1.42)	(0.91–1.94)
Level of Education	High School	1.07	0.59	0.92	0.83	1.23
		(0.83–1.37)	(0.50–0.70)***	(0.70–1.20)	(0.50–1.30)	(0.88–1.68)
	College	0.95	1.08	1.00	1.55	1.29
		(0.73–1.22)	(0.94–1.25)	(0.77–1.29)	(1.06–2.22)*	(0.94–1.75)
	University	Ref	Ref	Ref	Ref	Ref
Wealth Index	Quintile 1	1.16	0.89	1.61	1.58	1.15
		(0.87–1.55)	(0.75–1.06)	(1.16–2.24)**	(0.97–2.62)	(0.79–1.67)
	Quintile 2	1.61	1.68	2.87	1.56	1.27
		(1.23–2.11)***	(1.44–1.97)***	(2.14–3.91)***	(0.96–2.60)	(0.88–1.84)
	Quintile 3	1.02	0.89	1.48	1.54	0.88
		(0.76–1.37)	(0.75–1.06)	(1.06–2.07)*	(0.94–2.57)	(0.59–1.32)
	Quintile 4	0.69	0.61	0.64	0.76	1.01
		(0.50–0.95)*	(0.50–0.73)***	(0.43–0.96)*	(0.42–1.36)	(0.69–1.48)
	Quintile 5	Ref	Ref	Ref	Ref	Ref

*Ref.* Reference category, *O.R* Odds Ratio, *C.I* Confidence Interval

^*^p < 0.05, **p < 0.01, ***p < 0.001

^a^Results from a univariatelogisticregressionmodel

**Table 3:** Independent socio-demographic predictors of current substance use^a^

Variable	Category	Tobacco Current	Alcohol Current	Cannabis Current	Sedatives Current	Khat/Amphetamines Current
		**A.O.R (95% C.I)**	**A.O.R (95% C.I)**	**A.O.R (95% C.I)**	**A.O.R (95% C.I)**	**A.O.R (95% C.I)**
Sex	Male	3.74	1.87	2.89	0.99	2.69
		(2.99–4.71)***	(1.67–2.10)***	(2.32–3.62)***	(0.72–1.36)	(2.05–3.57)***
	Female	Ref	Ref	Ref	Ref	Ref
Age	Mean(SD)	1.08	1.08	1.06	1.09	1.1
		(1.04–1.13)***	(1.05–1.10)***	(1.01–1.10)**	(1.02–1.17)**	(1.05–1.15)***
Marital status	Married	Ref	Ref	Ref	Ref	Ref
	Single	1.33	1.22	1.27	2.06	0.85
		(0.87–2.15)	(0.96–1.57)	(0.81–2.09)	(0.99–5.06)	(0.54–1.42)
	Others	5.77	2.91	2.62	2.33	2.91
		(2.20–14.0)***	(1.39–5.93)**	(0.72–7.62)	(0.12–13.8)	(0.81–8.33)
Religion	Protestant	Ref	Ref	Ref	Ref	Ref
	Catholic	1.13	1.25	1.05	1.05	1.24
		(0.93–1.37)	(1.12–1.41)***	(0.85–1.29)	(0.75–1.46)	(0.96–1.59)
	Muslim	1.02	0.50	1.03	1.29	1.38
		(0.64–1.55)	(0.36–0.69)***	(0.64–1.57)	(0.62–2.39)	(0.79–2.26)
	Others	0.77	0.78	1.35	0.81	0.89
		(0.43–1.27)	(0.57–1.05)	(0.84–2.08)	(0.28–1.82)	(0.42–1.68)
Birth order	1–2	Ref	Ref	Ref	Ref	Ref
	3–5	0.84	0.90	0.82	1.06	1.03
		(0.68–1.02)	(0.80–1.01)	(0.66–1.01)	(0.76–1.47)	(0.79–1.34)
	6 +	0.67	0.67	0.60	0.82	1.31
		(0.45–0.95)*	(0.54–0.83)***	(0.39–0.89)*	(0.44–1.43)	(0.88–1.90)
Level of Education	High School	1.14	0.65	1.01	0.98	1.3
		(0.87–1.46)	(0.55–0.78)***	(0.75–1.34)	(0.58–1.57)	(0.93–1.80)
	College	0.95	1.10	1.02	1.54	1.26
		(0.73–1.23)	(0.95–1.27)	(0.78–1.32)	(1.05–2.22)*	(0.91–1.71)
	University	Ref	Ref	Ref	Ref	Ref
Wealth Index	Quintile 1	1.17	0.92	1.62	1.58	1.17
		(0.88–1.57)	(0.77–1.09)	(1.17–2.26)**	(0.96–2.62)	(0.80–1.70)
	Quintile 2	1.79	1.81	3.12	1.54	1.40
		(1.36–2.36)***	(1.54–2.12)***	(2.32–4.26)***	(0.94–2.56)	(0.97–2.04)
	Quintile 3	1.03	0.90	1.51	1.51	0.87
		(0.76–1.39)	(0.75–1.07)	(1.08–2.12)*	(0.92–2.52)	(0.58–1.30)
	Quintile 4	0.69	0.61	0.65	0.75	1.00
		(0.49–0.95)*	(0.51–0.74)***	(0.43–0.97)*	(0.41–1.35)	(0.68–1.47)
	Quintile 5	Ref	Ref	Ref	Ref	Ref

*Ref.* Reference category, *A.O.R* Adjusted Odds Ratio, *C.I* Confidence Interval

^*^p < 0.05, **p < 0.01, ***p < 0.001

^a^Results from a multivariatelogisticregression model

Tobacco use was associated with sex, age, marital status, birth order and wealth index. Alcohol use was associated with sex, age, marital status, religion, birth order, education level and wealth index. Cannabis use was associated with sex, age,birth order and wealth index.

Sedatives use was associated with age, education level and wealth index. Khat/amphetamine use was associated with sex and age.

Tobacco use was significantly predicted by male sex, older age, not being married /notsingle, being 6th born and above in the family, and wealth index Quintile 2 and Quintile 4. Alcohol use was significantly predicted by male sex, older age, not being married /notsingle, being a catholic, being a Muslim, higher sibling birth-order (6 +), high school students and wealth index Quintile 2 and Quintile 4. Cannabis use was significantly predicted by male sex, older age, higher sibling birth-order (6 +) and wealth index (Quintile 1 to Quintile 4). Sedatives use was significantly predicted by age and college students. Khat/amphetamine use was significantly predicted by male sex and older age.

### Economic factors associated with current substance abuse and independent economic predictors of current substance abuse.

These are summarized in Tables 4 and 5. Significant (*p* < 0.05) associations are highlighted for quick reference. More information on socio-economic characteristics disaggregated by current alcohol use are summarized in supplementary table 2.

**Table 4:** Economic indicators associated with current substance use^a^

Variable	Category	Tobacco Current	Alcohol Current	Cannabis Current	Sedatives Current	Khat/Amphetamine Current
		**O.R (95% C.I)**	**O.R (95% C.I)**	**O.R (95% C.I)**	**O.R (95% C.I)**	**O.R (95% C.I)**
Electricity	No	Ref	Ref	Ref	Ref	Ref
	Yes	2.04	2.44	3.03	1.88	1.35
		(1.66–2.54)***	(2.15–2.76)***	(2.37–3.91)***	(1.33–2.72)***	(1.05–1.75)*
Radio	No	Ref	Ref	Ref	Ref	Ref
	Yes	1.34	1.47	0.98	1.30	0.98
		(1.04–1.75)*	(1.26–1.71)***	(0.77–1.26)	(0.86–2.06)	(0.73–1.35)
Television	No	Ref	Ref	Ref	Ref	Ref
	Yes	1.75	2.07	2.14	1.43	1.10
		(1.43–2.14)***	(1.85–2.34)***	(1.73–2.68)***	(1.04–2.00)*	(0.87–1.41)
Refrigerator	No	Ref	Ref	Ref	Ref	Ref
	Yes	1.89	2.13	2.63	1.39	1.37
		(1.57–2.28)***	(1.91–2.39)***	(2.17–3.18)***	(0.99–1.92)	(1.06–1.77)*
Cell phone	No	Ref	Ref	Ref	Ref	Ref
	Yes	1.33	1.78	1.80	1.53	0.91
		(1.07–1.66)*	(1.55–2.04)***	(1.40–2.34)***	(1.04–2.31)*	(0.70–1.20)
Bicycle	No	Ref	Ref	Ref	Ref	Ref
	Yes	1.52	1.29	1.22	1.23	1.25
		(1.27–1.82)***	(1.16–1.44)***	(1.01–1.47)*	(0.90–1.66)	(0.99–1.58)
Motorcycle	No	Ref	Ref	Ref	Ref	Ref
	Yes	1.33	1.05	0.93	1.28	1.40
		(1.08–1.63)**	(0.92–1.20)	(0.73–1.17)	(0.89–1.81)	(1.06–1.82)*
Motor vehicle	No	Ref	Ref	Ref	Ref	Ref
	Yes	1.66	1.79	1.93	1.15	1.16
		(1.35–2.02)***	(1.59–2.02)***	(1.57–2.37)***	(0.78–1.64)	(0.87–1.54)
Source of water	Piped water	Ref	Ref	Ref	Ref	Ref
	Public water	0.87	0.55	0.70	0.70	0.93
		(0.66–1.13)	(0.46–0.65)***	(0.53–0.92)*	(0.42–1.13)	(0.65–1.30)
	Well water	0.67	0.62	0.51	0.84	0.71
		(0.53–0.84)***	(0.54–0.70)***	(0.40–0.64)***	(0.58–1.20)	(0.53–0.96)*
	Surface water	0.54	0.41	0.32	0.55	0.50
		(0.42–0.69)***	(0.35–0.48)***	(0.24–0.42)***	(0.35–0.84)**	(0.35–0.70)***
	Other source	0.60	0.44	0.31	0.26	0.62
		(0.25–1.19)	(0.27–0.69)***	(0.09–0.74)*	(0.01–1.20)	(0.19–1.50)
Earth floor	No	Ref	Ref	Ref	Ref	Ref
	Yes	0.63	0.51	0.36	0.70	0.65
		(0.49–0.80)***	(0.44–0.59)***	(0.26–0.48)***	(0.46–1.03)	(0.46–0.88)**
Cement floor	No	Ref	Ref	Ref	Ref	Ref
	Yes	0.92	1.03	0.99	1.00	1.11
		(0.77–1.10)	(0.93–1.14)	(0.82–1.20)	(0.73–1.35)	(0.88–1.42)
Tile floor	No	Ref	Ref	Ref	Ref	Ref
	Yes	1.63	1.80	2.09	1.31	1.13
		(1.33–1.99)***	(1.60–2.04)***	(1.71–2.56)***	(0.91–1.86)	(0.84–1.50)
Wood floor	No	Ref	Ref	Ref	Ref	Ref
	Yes	1.41	0.76	0.93	1.62	1.55
		(0.76–2.40)	(0.48–1.14)	(0.42–1.78)	(0.57–3.61)	(0.69–2.98)
Other floor material	No	Ref	Ref	Ref	Ref	Ref
	Yes	0.61	0.72	0.68	0.00	0.00
		(0.03–2.87)	(0.21–1.84)	(0.04–3.20)	(0.00–10,309)	(0.00–3.12)
Toilet	No toilet	Ref	Ref	Ref	Ref	Ref
	Pit latrine	0.47	0.99	0.51	0.33	0.55
		(0.27–0.88)*	(0.65–1.59)	(0.28–1.05)*	(0.16–0.86)*	(0.27–1.31)
	Flush toilet	0.89	1.78	1.27	0.69	0.81
		(0.51–1.68)	(1.15–2.88)*	(0.69–2.61)	(0.32–1.81)	(0.40–1.96)
	Other toilet facility	0.81	0.83	0.68	0.31	0.35
		(0.38–1.76)	(0.46–1.50)	(0.28–1.67)	(0.06–1.18)	(0.09–1.18)
Cooking method	Firewood	Ref	Ref	Ref	Ref	Ref
	Charcoal	1.43	1.16	1.89	1.37	1.14
		(1.07–1.87)*	(0.98–1.38)	(1.37–2.58)***	(0.82–2.21)	(0.78–1.62)
	Kerosene stove	1.95	1.38	2.22	2.17	1.35
		(1.24–2.95)**	(1.02–1.83)*	(1.29–3.59)**	(1.00–4.18)*	(0.70–2.36)
	Gas stove	2.13	2.42	3.84	2.06	1.26
		(1.74–2.61)***	(2.14–2.72)***	(3.07–4.83)***	(1.45–2.95)***	(0.95–1.67)
	Electric stove	1.92	2.42	5.65	4.76	2.88
		(1.13–3.09)*	(1.80–3.21)***	(3.67–8.46)***	(2.53–8.38)***	(1.67–4.67)***
	Other	1.70	2.02	4.47	0.74	1.78
		(0.71–3.44)	(1.27–3.11)**	(2.21–8.19)***	(0.04–3.41)	(0.62–4.02)

*Ref.* Reference category, *O.R* Odds Ratio, *C.I* Confidence Interval

^*^p < 0.05, **p < 0.01, ***p < 0.001

^a^Results from a univariatelogisticregressionmodel

**Table 5:** Independent economic indicators that predict current substance use^a^

Variable	Category	Tobacco Current	Alcohol Current	Cannabis Current	Sedatives Current	Khat/Amphetamine Current
		**A.O.R (95% CI)**	**A.O.R (95% CI)**	**A.O.R (95% CI)**	**A.O.R (95% CI)**	**A.O.R (95% CI)**
Electricity	No	Ref	Ref	Ref	Ref	Ref
	Yes	1.54	1.53	1.66	1.62	1.25
		(1.17–2.05)**	(1.30–1.80)***	(1.21–2.29)**	(1.01–2.61)*	(0.89–1.75)
Radio	No	Ref	Ref	Ref	Ref	Ref
	Yes	1.07	1.11	0.74	1.22	1.01
		(0.81–1.42)	(0.94–1.31)	(0.56–0.99)*	(0.77–2.00)	(0.72–1.43)
Television	No	Ref	Ref	Ref	Ref	Ref
	Yes	1.02	1.13	1.02	0.87	0.75
		(0.78–1.35)	(0.97–1.33)	(0.76–1.37)	(0.56–1.37)	(0.54–1.06)
Refrigerator	No	Ref	Ref	Ref	Ref	Ref
	Yes	1.13	1.19	1.38	0.82	1.15
		(0.87–1.45)	(1.02–1.39)*	(1.06–1.78)*	(0.52–1.27)	(0.81–1.64)
Cell phone	No	Ref	Ref	Ref	Ref	Ref
	Yes	1.02	1.33	1.36	1.32	0.81
		(0.80–1.29)	(1.15–1.55)***	(1.04–1.80)*	(0.88–2.04)	(0.61–1.09)
Bicycle	No	Ref	Ref	Ref	Ref	Ref
	Yes	1.40	1.14	1.17	1.22	1.26
		(1.15–1.72)***	(1.01–1.28)*	(0.94–1.45)	(0.86–1.71)	(0.96–1.64)
Motorcycle	No	Ref	Ref	Ref	Ref	Ref
	Yes	1.06	0.80	0.72	1.21	1.33
		(0.84–1.32)	(0.69–0.92)**	(0.55–0.93)*	(0.82–1.76)	(0.99–1.78)
Motor vehicle	No	Ref	Ref	Ref	Ref	Ref
	Yes	0.98	1.03	0.97	0.68	0.81
		(0.76–1.25)	(0.89–1.20)	(0.75–1.25)	(0.43–1.06)	(0.57–1.15)
Source of water	Piped water	Ref	Ref	Ref	Ref	Ref
	Public water	1.13	0.75	1.04	0.85	0.98
		(0.85–1.49)	(0.63–0.90)**	(0.78–1.39)	(0.50–1.40)	(0.68–1.40)
	Well water	0.88	0.83	0.79	1.10	0.77
		(0.69–1.12)	(0.72–0.95)**	(0.61–1.01)	(0.74–1.63)	(0.56–1.05)
	Surface water	0.85	0.67	0.67	0.85	0.57
		(0.64–1.12)	(0.57–0.79)***	(0.49–0.92)*	(0.52–1.37)	(0.39–0.83)**
	Other source	0.70	0.58	0.38	0.34	0.67
		(0.29–1.43)	(0.35–0.92)*	(0.12–0.93)	(0.02–1.59)	(0.20–1.65)
Earth floor	No	Ref	Ref	Ref	Ref	Ref
	Yes	0.81	0.81	0.52	0.60	0.16
		(0.31–1.95)	(0.46–1.38)	(0.18–1.43)	(0.12–2.90)	(0.06–0.52)**
Cement floor	No	Ref	Ref	Ref	Ref	Ref
	Yes	0.77	0.90	0.69	0.54	0.21
		(0.30–1.82)	(0.52–1.52)	(0.24–1.84)	(0.11–2.52)	(0.08–0.66)**
Tile floor	No	Ref	Ref	Ref	Ref	Ref
	Yes	0.80	0.93	0.68	0.44	0.17
		(0.30–1.93)	(0.53–1.58)	(0.23–1.82)	(0.09–2.11)	(0.06–0.57)**
Wood floor	No	Ref	Ref	Ref	Ref	Ref
	Yes	1.21	0.85	0.66	1.01	0.29
		(0.39–3.35)	(0.42–1.66)	(0.18–2.17)	(0.16–5.81)	(0.08–1.11)
Other floor material	No	Ref	Ref	Ref	Ref	Ref
	Yes	0.60	0.82	0.68	0.00	0.00
		(0.03–3.57)	(0.22–2.42)	(0.03–4.41)	(0.00–0.00)	(0.00–0.00)
Toilet	No toilet	Ref	Ref	Ref	Ref	Ref
	Pit latrine	0.39	0.70	0.37	0.26	0.54
		(0.22–0.75)**	(0.45–1.15)	(0.19–0.79)**	(0.12–0.70)**	(0.26–1.32)
	Flush toilet	0.47	0.63	0.37	0.42	0.66
		(0.26–0.93)*	(0.39–1.05)	(0.18–0.80)**	(0.17–1.18)	(0.30–1.68)
	Other toilet facility	0.70	0.68	0.57	0.27	0.33
		(0.33–1.54)	(0.37–1.25)	(0.23–1.44)	(0.06–1.06)	(0.08–1.13)
Cooking method	Firewood	Ref	Ref	Ref	Ref	Ref
	Charcoal	1.20	0.91	1.42	1.28	0.99
		(0.89–1.60)	(0.76–1.09)	(1.01–1.97)*	(0.75–2.11)	(0.67–1.43)
	Kerosene stove	1.68	1.13	1.65	1.93	1.16
		(1.05–2.57)*	(0.84–1.52)	(0.95–2.70)	(0.87–3.82)	(0.60–2.07)
	Gas stove	1.49	1.54	2.28	1.79	0.98
		(1.13–1.95)**	(1.32–1.80)***	(1.71–3.05)***	(1.12–2.85)*	(0.68–1.41)
	Electric stove	1.21	1.52	3.16	4.32	2.15
		(0.68–2.06)	(1.10–2.09)*	(1.94–5.04)***	(2.06–8.62)***	(1.16–3.84)*
	Other	1.13	1.64	2.91	0.55	1.35
		(0.46–2.38)	(1.01–2.58)*	(1.39–5.54)**	(0.03–2.67)	(0.46–3.18)

*Ref.* Reference category, *A.O.R* Adjusted Odds Ratio, *C.I* Confidence Interval

^*^p < 0.05, **p < 0.01, ***p < 0.001

^a^Results from a multivariatelogisticregression model

Tobacco use was associated with all the economic indicators except household having cement floor, wood floor and other floor material. Alcohol use was associated with all the economic indicators except household having motorcycle, cement floor, wood floor and other floor material. Cannabis use was associated with all the economic indicators except household having radio, motorcycle, cement floor, wood floor and other floor material.

Sedatives use was associated with household having electricity, television, cell phone, sources of water, toilet and cooking method. Sedatives use was associated with household having electricity, television, cell phone, sources of water, toilet and cooking method. Khat/amphetamine use was associated with household having electricity, refrigerator, motorcycle, sources of water, earth floor, toilet and cooking method.

Tobacco use was significantly predicted by household having electricity, bicycle, pit latrine, flush toilet and cooking method with kerosene stove and gas stove. Alcohol use was significantly predicted by household having electricity, refrigerator, cellphone, bicycle, public water, well water, surface water, other sources of water and cooking method with gas stove, electric stove and other methods. Cannabis use was significantly predicted by household having electricity, radio, refrigerator, cellphone, motorcycle, surface water, pit latrine, flush toilet and cooking method with charcoal, gas stove, electric stove and other methods. Sedatives use was significantly predicted by household having electricity, pit latrine and cooking method with gas stove and electric stove. Khat/amphetamine use was significantly predicted by household having surface water, earthfloor, cement floor, tile floor and cooking method with electric stove.

### Psychiatric disorders and substance abuse

Table 6 summarizes the crude associations between psychiatric disorders and current substance use with significant associations highlighted for quick reference. There is highly significant association between all types of substance use disorders and most psychiatric disorders. There are just a few exceptions (*p* > 0.05) for specific substance abuse, namely bulimia and psychosisfor alcohol; panic disorder for cannabis; agoraphobia for alcohol, cannabis and amphetamine/khat.

**Table 6:** Crude association between psychiatric disorders and current substance use^a^

PDSQ Disorders	Tobacco Current		Alcohol Current		Cannabis Current		Sedatives Current		Amphetamines/Khat Current	
	O.R	Sig	O.R	Sig	O.R	Sig	O.R	Sig	O.R	Sig
	(95% C.I)		(95% C.I)		(95% C.I)		(95% C.I)		(95% C.I)	
Major Depressive Disorder	2.02	** < 0.001**	1.37	** < 0.001**	1.72	** < 0.001**	2.61	** < 0.001**	1.53	**0.001**
	(1.67–2.43)		(1.22–1.55)		(1.40–2.10)		(1.91–3.54)		(1.17–1.97)	
PTSD	1.46	** < 0.001**	1.22	** < 0.001**	1.32	**0.006**	2.39	** < 0.001**	1.58	** < 0.001**
	(1.21–1.75)		(1.09–1.36)		(1.08–1.61)		(1.76–3.24)		(1.23–2.01)	
Bulimia/Binge Eating Disorder	1.83	**0.003**	1.19	0.22	1.67	**0.019**	2.52	**0.002**	2.02	**0.005**
	(1.21–2.66)		(0.89–1.56)		(1.06–2.50)		(1.35–4.31)		(1.20–3.21)	
OCD	1.36	**0.002**	1.17	**0.006**	1.29	**0.015**	1.92	** < 0.001**	1.5	**0.002**
	(1.12–1.66)		(1.05–1.30)		(1.05–1.58)		(1.35–2.80)		(1.16–1.97)	
Panic Disorder	1.52	** < 0.001**	1.14	**0.048**	1.18	0.145	2.1	** < 0.001**	1.83	** < 0.001**
	(1.24–1.86)		(1.00–1.29)		(0.94–1.47)		(1.52–2.89)		(1.41–2.36)	
Psychosis	1.69	** < 0.001**	1.1	0.077	1.32	**0.004**	2.06	** < 0.001**	1.36	**0.01**
	(1.41–2.01)		(0.99–1.22)		(1.09–1.59)		(1.52–2.82)		(1.07–1.72)	
Agoraphobia	1.35	** < 0.001**	1.11	0.069	1.01	0.889	1.78	** < 0.001**	1.26	0.062
	(1.13–1.62)		(0.99–1.23)		(0.83–1.23)		(1.31–2.41)		(0.99–1.59)	
Social Phobia	1.45	** < 0.001**	1.45	** < 0.001**	1.41	** < 0.001**	2.51	** < 0.001**	1.74	** < 0.001**
	(1.22–1.74)		(1.31–1.61)		(1.17–1.70)		(1.81–3.54)		(1.37–2.22)	
Alcohol Abuse/Dependence	5.32	** < 0.001**	3.21	** < 0.001**	4.54	** < 0.001**	2.36	** < 0.001**	3.14	** < 0.001**
	(4.45–6.38)		(2.87–3.59)		(3.76–5.49)		(1.73–3.21)		(2.48–3.98)	
Drug Abuse/Dependence	6.01	** < 0.001**	2.68	** < 0.001**	5.48	** < 0.001**	2.4	** < 0.001**	3.52	** < 0.001**
	(5.02–7.20)		(2.37–3.02)		(4.53–6.63)		(1.72–3.30)		(2.77–4.47)	
Generalized Anxiety Disorder	1.56	** < 0.001**	1.6	** < 0.001**	1.34	**0.021**	2.36	** < 0.001**	1.76	** < 0.001**
	(1.23–1.94)		(1.39–1.84)		(1.04–1.71)		(1.66–3.31)		(1.31–2.33)	
Somatization Disorder	1.52	** < 0.001**	1.19	**0.004**	1.39	**0.001**	2.41	** < 0.001**	1.58	** < 0.001**
	(1.25–1.82)		(1.06–1.33)		(1.14–1.70)		(1.77–3.27)		(1.24–2.02)	
Hypochondriasis	1.73	** < 0.001**	1.27	** < 0.001**	1.5	** < 0.001**	1.84	** < 0.001**	1.9	** < 0.001**
	(1.44–2.07)		(1.14–1.43)		(1.23–1.82)		(1.34–2.50)		(1.49–2.40)	
Suicidality	1.81	** < 0.001**	1.38	** < 0.001**	1.54	** < 0.001**	2.41	** < 0.001**	1.55	** < 0.001**
	(1.50–2.19)		(1.23–1.56)		(1.26–1.89)		(1.77–3.28)		(1.20–1.99)	

*O.R* Odds Ratio, *C.I* Confidence Interval

^a^Results from a univariatelogisticregressionmodel

Table 7 summarizes the independent psychiatric predictors of substance abuse. Significant (*p* < 0.05) highlighted for quick reference.

**Table 7:** Independent psychiatric disorders that predict current substance use^a^

PDSQ Disorders	Tobacco Current		Alcohol Current		Cannabis Current		Sedatives Current		Amphetamines/Khat Current	
	A.O.R	Sig	A.O.R	Sig	A.O.R	Sig	A.O.R	Sig	A.O.R	Sig
	(95% C.I)		(95% C.I)		(95% C.I)		(95% C.I)		(95% C.I)	
Major Depressive Disorder	1.43	**0.005**	1.08	0.370	1.40	**0.015**	1.28	0.238	0.90	0.542
	(1.11–1.85)		(0.92–1.27)		(1.06–1.83)		(0.85–1.91)		(0.64–1.26)	
PTSD	0.79	0.052	0.93	0.333	0.86	0.233	1.36	0.103	1.04	0.781
	(0.63–1.00)		(0.81–1.07)		(0.67–1.10)		(0.94–1.96)		(0.77–1.40)	
Bulimia/Binge Eating Disorder	0.92	0.700	0.80	0.147	1.07	0.790	1.13	0.700	1.17	0.569
	(0.59–1.39)		(0.59–1.08)		(0.65–1.67)		(0.58–2.03)		(0.67–1.94)	
OCD	0.9	0.373	0.98	0.742	0.99	0.947	1.03	0.877	1.07	0.649
	(0.71–1.14)		(0.86–1.12)		(0.78–1.27)		(0.68–1.60)		(0.79–1.47)	
Panic Disorder	0.94	0.621	0.86	0.059	0.81	0.128	1.03	0.870	1.31	0.094
	(0.73–1.21)		(0.73–1.01)		(0.61–1.06)		(0.69–1.53)		(0.95–1.80)	
Psychosis	1.00	0.970	0.73	** < 0.001**	0.80	0.075	1.07	0.737	0.73	**0.040**
	(0.79–1.26)		(0.64–0.84)		(0.63–1.02)		(0.73–1.58)		(0.54–0.98)	
Agoraphobia	0.76	**0.024**	0.77	** < 0.001**	0.58	** < 0.001**	0.83	0.323	0.68	**0.011**
	(0.61–0.96)		(0.67–0.89)		(0.45–0.74)		(0.57–1.20)		(0.50–0.91)	
Social Phobia	0.86	0.196	1.24	**0.001**	1.05	0.694	1.57	**0.029**	1.27	0.122
	(0.68–1.08)		(1.09–1.42)		(0.83–1.33)		(1.05–2.36)		(0.94–1.71)	
Alcohol Abuse/Dependence	3.14	** < 0.001**	3.05	** < 0.001**	2.80	** < 0.001**	1.34	0.166	1.93	** < 0.001**
	(2.45–4.03)		(2.63–3.54)		(2.16–3.64)		(0.88–2.03)		(1.39–2.66)	
Drug Abuse/Dependence	3.52	** < 0.001**	1.61	** < 0.001**	3.84	** < 0.001**	1.30	0.234	2.25	** < 0.001**
	(2.76–4.50)		(1.37–1.88)		(2.96–4.97)		(0.84–2.00)		(1.63–3.12)	
Generalized Anxiety Disorder	1.33	**0.033**	1.67	** < 0.001**	1.24	0.147	1.36	0.123	1.37	0.063
	(1.02–1.73)		(1.41–1.96)		(0.92–1.64)		(0.91–2.01)		(0.98–1.89)	
Somatization Disorder	0.91	0.404	0.89	0.113	0.96	0.732	1.54	**0.019**	1.00	0.992
	(0.72–1.14)		(0.77–1.03)		(0.75–1.22)		(1.07–2.20)		(0.74–1.33)	
Hypochondriasis	0.85	0.183	0.85	**0.034**	0.83	0.143	0.79	0.222	1.15	0.375
	(0.67–1.08)		(0.74–0.99)		(0.65–1.06)		(0.54–1.15)		(0.85–1.55)	
Suicidality	1.02	0.873	1.06	0.468	0.94	0.651	1.37	0.104	1.04	0.822
	(0.81–1.28)		(0.91–1.22)		(0.73–1.21)		(0.94–1.98)		(0.76–1.40)	

*A.O.R* Adjusted Odds Ratio, *C.I* Confidence Interval

^a^Results from a multivariatelogisticregression model

Tobacco use was significantly predicted by major depressive disorder, agoraphobia, alcohol abuse/dependence, drug abuse/dependence and generalized anxiety disorder. Alcohol use was significantly predicted by psychosis, agoraphobia, social phobia, alcohol abuse/dependence, drug abuse/dependence and generalized anxiety disorder. Cannabis use was significantly predicted by major depressive disorder, agoraphobia, alcohol abuse/dependence and drug abuse/dependence. Sedatives use was significantly predicted by social phobia and somatization disorder. Khat/amphetamine use was significantly predicted by psychosis, agoraphobia, alcohol abuse/dependence and drug abuse/dependence.

## Discussion

Overall, we present a Kenyan study that has several strengths over and above previously reported Kenyan studies. These include a large sample spanning different levels of education, different associations and predictors of alcohol and drug abuse all conducted concurrently in the study participants. Our discussion is aligned to the specific aims of this study and what the findings imply for public awareness and clinical interventions.

We have demonstrated the occurrence of a wide range of substance abuse and in the process confirmed earlier trends and in particular those by NACADA using nationally representative sample. However, we did find a higher level of alcohol (17.7%) of current use compared with 12.2% by NACADA. This would suggest a higher vulnerability in students compared with the general population, thus the need for interventional programs specifically focused in educational institutions. Of special note is that tobacco, with well known addictive properties and long-term medical complications, was the second most prevalent substance of abuse after alcohol.Itsaddictive potential can be discerned from our data in that, the drop in use between lifetime, previous 3 months and current is much smaller thanwith alcohol. This finding on tobacco can be understood to some extent in the Kenyan context. Unlike alcohol, which is easily affordable and widely available, cigarettes are much more expensive. Their availability is highly restricted by law in that retailing is limited to a pack of 20 rather than single sticks of cigarettes. It is possible that even if the retail outlets only allowed packet of 20, people or their agents could still pool resources for one of them to by a packet of 20 and then share and in the process circumvent the legal requirements not to sell single cigarette. Cannabis is widely available. Its trafficking, though prohibited by law, still occurs. In the Kenyan context the psycho-stimulant khat which acts like amphetamines is widely available and lawful. Growing and marketing of khat is allowed by the Government. Although WHO classifies khat as a drug of abuse, in the Kenyan context khat presents a dilemma in that farmers who grow it and make a living from it are also voters whom politicians want to appease. Of concern are the sedatives which users of psycho-stimulants use to bring them down. Therefore, the similarity in the use of khat/amphetamine and sedatives is not surprising. But unlike khat they are prescription drugs. Obviously they are being obtained through fraudulent prescriptions or unscrupulous pharmacies. Hallucinogens, Cocaine and Opioids, though still at very low prevalence possibly because of high costs, are used by Kenyan youth. Inhalants from glue and petrol and which cost nothing to obtain, are associated with homeless street children, usually in urban areas. The epidemiological and prevalence patterns found in this study suggest a wide availability of different drugs and therefore the need for public awareness to minimize their availability in the population, reduce demand for themwhile at clinical level increased resources for interventions. In particular, there is need to revisit the policy on cigarette availability, the need for more tight regulations or prescriptions and the need to address khat production and marketing to alternative commercial activities in a way that does not antagonize the farmers.

Our findings suggest that both alcohol and substance abuse generate similar perceptions regarding drinking too much/using drugs too much and “thinking of cutting down” on the one hand and on the other hand “thinking that they had an alcohol drug problem at personal or social circles”. In other words, they do not perceive over-indulgence as a sign of a problem. The implication for this is the need for interventions that focus on the health problems both biological and psychosocial that can occur to the individual so that the individual can make an informed decision on overindulgence in these substances. A trend that emerges from our findings is that more students expressed negative perceptions about alcohol than other substances. A qualitative study may be needed to tease out the differences between alcohol and drug perceptions, and the explanations for those differences.

There were some interesting observations regarding the socio-demographics.It is not surprising that alcohol and substance abuse was more associated with the boys than girls. Boys more than girls tend to have more externalizing social behavior than girls [24]. Alcohol abuse and tobacco usestart early at high schools. This suggests that efforts to increase public health awareness should focus on high schools with the potential to avoid dependence that can continue to college and university. It is apparent that alcohol use was more in the university students and college students than in high school students. We speculate that alcohol use is an expression of independence when older groups are no longer under the direct supervision of parents as happens among high school students who are living at home. However, the common denominator in our findings is that these substances of abuse were found across the different levels of education calling for specific interventions directed at the different levels. All types of drugs were found across all religions.We replicated the common findings summarized in our literature review that alcohol use is more common in Catholics than in Protestants (from literature review). This has been explained as a result of tolerance to alcohol use in Catholics than in Protestants. However, we did not expect that Muslims would also use alcohol, which is strictly prohibited by their religion. This could be a reflection of changing trends towards more liberal attitudes facilitated by Muslim students interacting with their non-Muslim peers. The fact that most of them were either first or second born is a reflection of the increasingly small families in Kenya. However, tobacco and alcohol use was higher in first or second borns than other birth orders. We have no immediate explanation for this.

There is a general caveat to the linking of household economic indicators to substance abuse. The wealth index for the households has its own limitation. Whereas our findings reflect the status at the time of data collection, the scenario is fast changing with increased electricity and water supply, (which go together with availability of flush toilets) as a result of government policies on highly subsidized electricity and piped water connection rather than a reflection of economic status. Further, some of the trends observed here based on household indicators may not apply to students. For example, availability of telephone in the household is superseded by the wide availability of highly affordable cell-phones, some of them smart phones. This allows them uncensored communication with their peers regardless of economic status of their families. Using that forum, they can share information on all kinds of drugs. This therefore suggests a need to use same forum for awareness of drugs and the need to involve them or their identified peer leaders in developing awareness campaigns. However, this does not rule out the impact of the family economic status on the students. With this caveat in mind, we can now discuss the findings in relation to economic indicators. Cannabis occurred mostly in the 3 lowest wealth index groups and least in the quintile 4, followed by quintile 5. We speculate the explanation for this is that cannabis is so widely available that access does not require substantial income. This is not so for khat/Amphetamines that seems to cut across all the age groups. However, more specific details emerge when specific indicators of wealth are considered. The finding that tobacco use was significantly (*p* < 0.05) associated with two indicators of low socio-economic status i.e. having no electricity in the house and use of pit latrine suggests wide availability of tobacco. The implication for this is that the policy of selling cigarettes in the more expensive packs rather than single sticks of cigarettes is not working or there are cheap brands available. Indeed it is possible to make one’s own cigarettes rolls of tobacco leaves from tobacco plants that are widely available. Aggressive marketing is also a possible contributor. Qualitative studies would provide more plausible explanations for the different economic indicators and their relevance if any to particular drug abuse in students and by extension youth outside the education system. Alcohol was found across different indicators of economic status ranging from availability of electricity, refrigerators, piped water on one hand to use of firewood and charcoal on the other hand. This suggests wide availability of alcohol across the spectrum of wealth and that cost is not an issue given the wide availability of cheap highly potent spirits in packages that costs about 20 US cents. Therefore the current policy not to pack alcohol in these sachets is not working. Further, traditional brew can cost as little as 10 US cents per unit of drink. Even though sedatives are used, they still remain technically prescription drugs and therefore relatively unavailable compared with other drugs regardless of socio-economic status. Regulations on sedatives as prescription drugs need to be enforced. Since khat/amphetamines and sedatives are used sequentially as already explained, it is not surprising that they are similar when it comes to socio-economic indicators. These findings call for review on existing policies on availability of different types of drugs while focusing on increasing public awareness that involves dialogue between different stakeholders including students in order to generate mutually acceptable change.

Our finding on co-morbidity of a wide range of psychiatric disorders has not been reported in Kenya before. They suggest that it is necessary to screen and manage any co-morbid psychiatric disorders, more so at clinical level. The independent psychiatric disorders that need special attention for public health awareness and clinical intervention at individual level are major depression and different types of anxiety disorders (agoraphobia and generalized anxiety disorder). The association between anxiety and or depression and alcohol or substance abuse could be a reflection of either use of these substances leading to psychiatric disorders, or these substances are used for self-medication. In the case of sedatives, it is possible that they are used to calm down after use of khat or any other psycho-stimulants. The association between psychosis and cannabis (*P* < 0.05) is widely reported in clinical practice.

### Potential for intervention

1. Interventions should start at high school

2. A public health approach to promote public awareness on different types of substance abuse. Emphasis should be on potential medical, psychological and social consequences of substances and in particular the addictive potential of cigarettes. This approach should go beyond the usual general warnings that cigarettes and alcohol are harmful, but without details regardinghow they are harmful. In particular, emphasis should be placed on psychiatric disorders that may complicate substance abuse and the need to address those disorders on their own right.

3. Public awareness should be matched withtraining of school-based counselors and training of personnel at institutional health facilities to provide integrated interventions that takes into account all the associated factors and in particular the association with various psychiatric disorders which for every student seeking help can be screened using PDSQ. The WHO mental health treatment gap-intervention guidelines have a section on substance abuse including alcohol and common psychiatric disorders that can be used by trained non-mental health specialists.

4. There is need for strict implementation of existing policies to restrict availability of branded but cheap alcohol and cigarettes to also include unbranded products.

## Conclusion

Different types of substance abuse and alcohol use are found in high schools, colleges and universities and start early at high school level. Easy availability at minimal or no costs at all seem to be the major driving factor. The students have different and varied perceptions on these substances. The use of these substances are associated with socio-demographic, economic and environmental indicators and psychiatric disorders. All of these need to be factored in public awareness and at clinical interventions. We have therefore achieved all the four aims summarized at the end of the Introduction.

### Limitations

There are several limitations in this study. Being across sectional quantitative study, it was not possible to establish directional associations between alcohol and substance abuse and associated factors. A qualitative approach would have enriched the quantitative data in understanding better the nuances between different types of drugs and alcohol abuse and associated factors. Although, the college and university students were drawn from all parts of the country through the process of centralized admission, we still did not have a predetermined sampling process that would ensure representations of all colleges and universities in Kenya. The findings in high school students remain unique only to those communities where the students were drawn from.

## Supplementary Information

**Additional file 1:Table 1.** Students Perceptions of their use of alcohol and substance abus. **Table 2.** Socio-economic characteristics disaggregated by Current alcohol use. **Table 3.** Given substance use associated with use of other substances††. **Table 4.** Independent predictors of a given substance use with other substance use. ┼┼. **Table 5.** Independent psychiatric disorders that predict current substance use adjusting for alcohol and substance dependence ┼┼.

## Data Availability

The datasets used and/or analyzedare available from the corresponding author on reasonable request.

## Abbreviations

DSM-IV: Diagnostic and Statistical Manual of Mental Disorders, Fourth Edition
NACADA: National Authority for the Campaign Against Alcohol and Drug Abuse
LMIC: Low and Middle Income Countries
ASSIST: Alcohol, Smoking, and Substance Involvement Screening Test
WHO: World Health Organization
PDSQ: Psychiatric Diagnostic Screening Questionnaire
AMHRTF: Africa Mental Health Research and Training Foundation
