# Supplementary material for: Prevalence and perception of substance abuse and associated economic indicators and mental health disorders in a large cohort of Kenyan students: towards integrated public health approach and clinical management
Source: BMC Psychiatry. 2022 Mar 17;22:191. doi: 10.1186/s12888-022-03817-2 (PMC8932181; doi:10.1186/s12888-022-03817-2)
Supplement: Supplementary file 1 — Additional file 1:Table 1. Students Perceptions of their use of alcohol and substance abus. Table 2. Socio-economic characteristics disaggregated by Current alcohol use. Table 3. Given substance use associated with use of other substances††. Table 4. Independent predictors of a given substance use with other substance use. ┼┼. Table 5. Independent psychiatric disorders that predict current substance use adjusting for alcohol and substance dependence ┼┼. [file 12888_2022_3817_MOESM1_ESM.doc]

**SUPPLEMENTARY TABLES**

Table 1: Students Perceptions of their use of alcohol and substance abuse

| **Alcohol Abuse**  **(PDSQ)** | **Frequency**  **(N=9742)** | **Percentage**  **(%)** | **Missing** |
| --- | --- | --- | --- |
| Did you think that you were drinking too much? | 1091 | 11.2 | 13 |
| Did anyone in your family think or say that you were drinking too much, or that you had an alcohol problem? | 810 | 8.3 | 11 |
| Did friends, a doctor, or anyone else think or say that you were drinking too much? | 795 | 8.2 | 5 |
| Did you think about cutting down or limiting you’re drinking? | 1313 | 13.5 | 12 |
| Did you think that you had an alcohol problem? | 698 | 7.2 | 17 |
| Because of your drinking did you have problems in your marriage; at your job; with your friends or family; doing household chores; or in any other important area of your life? | 725 | 7.4 | 8 |
| **Alcohol Abuse/Dependence:** | **2198** | **22.6** | 1 |

| **Substance Abuse**  **(PDSQ)** | **Frequency**  **(N=9742)** | **Percentage**  **(%)** | **Missing** |
| --- | --- | --- | --- |
| Did you think that you were using drugs too much? | 686 | 7.0 | 10 |
| Did anyone in your family think or say that you were using drugs too much, or that you had a drug problem? | 635 | 6.5 | 14 |
| Did friends, a doctor, or anyone else think or say that you were using drugs too much? | 648 | 6.7 | 5 |
| Did you think about cutting down or limiting your drug use? | 1015 | 10.4 | 10 |
| Did you think that you had a drug problem? | 611 | 6.3 | 14 |
| Because of your drug use did you have problems in your marriage; at your job; with your friends or family; doing household chores; or in any other important area of your life? | 640 | 6.6 | 16 |
| **Drug Abuse/Dependence:** | **1670** | **17.1** | **1** |

Note: This table summarizes the prevalence of different perceptions of alcohol and substance abuse.

Table 2: Socio-economic characteristics disaggregated by Current alcohol use

| **Items** | **Overall**  **(N=9742)** | **Current Alcohol Use** | | **P-Value** |
| --- | --- | --- | --- | --- |
|  |  | **No** | **Yes** |  |
| **Items in Household** | | | | |
| Electricity | 6208(63.7%) | 4852(60.5%) | 1356(78.8%) | **<0.001** |
| Radio | 8043(82.6%) | 6551(81.7%) | 1492(86.7%) | **<0.001** |
| Television | 5999(61.6%) | 4715(58.8%) | 1284(74.7%) | **<0.001** |
| Refrigerator | 2290(23.5%) | 1672(20.8%) | 618(35.9%) | **<0.001** |
| Cell phone | 7361(75.6%) | 5927(73.9%) | 1434(83.4%) | **<0.001** |
| Bicycle | 3847(39.5%) | 3081(38.4%) | 766(44.5%) | **<0.001** |
| Motorcycle | 1982(20.3%) | 1620(20.2%) | 362(21.0%) | 0.426 |
| Motor vehicle | 1892(19.4%) | 1415(17.6%) | 477(27.7%) | **<0.001** |
| **Homes source of water** | | | | |
| Piped water | 3087(31.7%) | 2329(29.2%) | 758(44.2%) | **<0.001** |
| Public water | 1332(13.7%) | 1130(14.2%) | 202(11.8%) |  |
| Well water | 2670(27.4%) | 2224(27.9%) | 446(26.0%) |  |
| Surface water | 2427(24.9%) | 2141(26.9%) | 286(16.7%) |  |
| Other source | 167(1.7%) | 146(1.8%) | 21(1.2%) |  |
| **Home Floor** | | | | |
| Earth floor | 2199(22.6%) | 1954(24.4%) | 245(14.2%) | **<0.001** |
| Cement floor | 5532(56.8%) | 4545(56.7%) | 987(57.4%) | 0.581 |
| Tile floor | 1843(18.9%) | 1376(17.2%) | 467(27.2%) | **<0.001** |
| Wood floor | 181(1.9%) | 156(1.9%) | 25(1.5%) | 0.171 |
| Other floor material | 30(0.3%) | 26(0.3%) | 4(0.2%) | 0.534 |
| **Homes toilet** | | | | |
| No toilet | 148(1.5%) | 125(1.6%) | 23(1.3%) | **<0.001** |
| Pit latrine | 7178(73.7%) | 6057(75.6%) | 1121(65.3%) |  |
| Flush toilet | 2173(22.3%) | 1631(20.4%) | 542(31.6%) |  |
| Other toilet facility | 231(2.4%) | 200(2.5%) | 31(1.8%) |  |
| **Household cooking method** | | | | |
| Firewood | 5075(52.1%) | 4409(55.0%) | 666(38.7%) | **<0.001** |
| Charcoal | 1339(13.7%) | 1139(14.2%) | 200(11.6%) |  |
| Kerosene stove | 348(3.6%) | 288(3.6%) | 60(3.5%) |  |
| Gas stove | 2607(26.8%) | 1908(23.8%) | 699(40.7%) |  |
| Electric stove | 256(2.6%) | 188(2.3%) | 68(4.0%) |  |
| Other | 111(1.1%) | 85(1.1%) | 26(1.5%) |  |

Note: This table summarizes the different associations between different economic indicators and current alcohol use, the most commonly used substance.

**Table 3: Given substance use associated with use of other substances**^††^

| **ASSIST Substance Use** | **Tobacco Current** | | **Alcohol Current** | | **Cannabis Current** | | **Sedatives Current** | | **Amphetamines/Khat**  **Current** | |
| --- | --- | --- | --- | --- | --- | --- | --- | --- | --- | --- |
|  | O.R | Sig. | O.R | Sig. | O.R | Sig. | O.R | Sig. | O.R | Sig. |
|  | (95% C.I) |  | (95% C.I) |  | (95% C.I) |  | (95% C.I) |  | (95% C.I) |  |
| Tobacco Current | — | — | 54.10 | **<0.001** | 28.70 | **<0.001** | 5.950 | **<0.001** | 10.40 | **<0.001** |
|  | — |  | (41.1, 72.8) |  | (23.1, 35.6) |  | (4.09, 8.47) |  | (7.97, 13.5) |  |
| Alcohol Current | 54.10 | **<0.001** | — | — | 38.00 | **<0.001** | 9.150 | **<0.001** | 6.09 | **<0.001** |
|  | (41.1, 72.8) |  | — |  | (29.3, 50.3) |  | (6.68, 12.7) |  | (4.81, 7.73) |  |
| Cannabis Current | 28.70 | **<0.001** | 38.00 | **<0.001** | — | — | 6.310 | **<0.001** | 9.61 | **<0.001** |
|  | (23.1, 35.6) |  | (29.3, 50.3) |  | — |  | (4.31, 9.05) |  | (7.28, 12.6) |  |
| Cocaine Current | 12.00 | **<0.001** | 9.55 | **<0.001** | 24.20 | **<0.001** | 25.300 | **<0.001** | 24.70 | **<0.001** |
|  | (7.19, 19.7) |  | (5.77, 16.2) |  | (14.8, 39.7) |  | (14.2, 43.5) |  | (14.8, 40.7) |  |
| Inhalants Current | 22.40 | **<0.001** | 15.40 | **<0.001** | 21.80 | **<0.001** | 33.100 | **<0.001** | 18.30 | **<0.001** |
|  | (13.1, 38.6) |  | (8.50, 30.0) |  | (12.7, 37.5) |  | (18.2, 58.4) |  | (10.2, 31.9) |  |
| Sedatives Current | 5.95 | **<0.001** | 9.15 | **<0.001** | 6.31 | **<0.001** | — | — | 13.50 | **<0.001** |
|  | (4.09, 8.47) |  | (6.68, 12.7) |  | (4.31, 9.05) |  | — |  | (9.33, 19.3) |  |
| Hallucinogens Current | 7.47 | **<0.001** | 6.23 | **<0.001** | 8.46 | **<0.001** | 38.20 | **<0.001** | 19.30 | **<0.001** |
|  | (4.53, 11.9) |  | (4.03, 9.71) |  | (5.12, 13.6) |  | (23.5, 61.2) |  | (11.9, 30.5) |  |
| Opiods Current | 14.00 | **<0.001** | 11.60 | **<0.001** | 23.40 | **<0.001** | 35.30 | **<0.001** | 33.00 | **<0.001** |
|  | (8.46, 23.1) |  | (6.88, 20.3) |  | (14.2, 38.6) |  | (20.4, 59.6) |  | (19.9, 54.6) |  |
| Amphetamines/Khat Current | 10.40 | **<0.001** | 6.09 | **<0.001** | 9.61 | **<0.001** | 13.50 | **<0.001** | — | — |
|  | (7.97, 13.5) |  | (4.81, 7.73) |  | (7.28, 12.6) |  | (9.33, 19.3) |  | — |  |

Note: ^††^ Results from a univariate logistic regression model; O.R = Odds Ratio; C.I = Confidence Interval

**Table 4: Independent predictors of a given substance use with other substance use. ^┼┼^**

| **ASSIST Substance Use** | **Tobacco Current** | | **Alcohol Current** | | **Cannabis Current** | | **Sedatives Current** | | **Amphetamines/Khat**  **Current** | |
| --- | --- | --- | --- | --- | --- | --- | --- | --- | --- | --- |
|  | A.O.R | Sig. | A.O.R | Sig. | A.O.R | Sig. | A.O.R | Sig. | A.O.R | Sig. |
|  | (95% C.I) |  | (95% C.I) |  | (95% C.I) |  | (95% C.I) |  | (95% C.I) |  |
| Tobacco Current | — | — | 30.9 | **<0.001** | 4.81 | **<0.001** | 0.88 | 0.598 | 3.02 | **<0.001** |
|  | — |  | (23.1, 42.1) |  | (3.77, 6.15) |  | (0.53, 1.42) |  | (2.10, 4.34) |  |
| Alcohol Current | 31.5 | **<0.001** | — | — | 19.6 | **<0.001** | 6.32 | **<0.001** | 2.41 | **<0.001** |
|  | (23.5, 42.8) |  | — |  | (14.7, 26.4) |  | (4.38, 9.14) |  | (1.75, 3.30) |  |
| Cannabis Current | 4.93 | **<0.001** | 19.2 | **<0.001** | — | — | 0.87 | 0.599 | 2.01 | **<0.001** |
|  | (3.86, 6.31) |  | (14.4, 26.0) |  | — |  | (0.51, 1.44) |  | (1.38, 2.92) |  |
| Cocaine Current | 1.03 | 0.932 | 1.33 | 0.506 | 5.15 | **<0.001** | 2.59 | **0.023** | 3.23 | **<0.001** |
|  | (0.48, 2.19) |  | (0.57, 3.01) |  | (2.51, 10.7) |  | (1.10, 5.68) |  | (1.60, 6.33) |  |
| Inhalants Current | 3.44 | **<0.001** | 1.39 | 0.483 | 2.02 | 0.063 | 4.89 | **<0.001** | 1.51 | 0.314 |
|  | (1.66, 7.24) |  | (0.56, 3.54) |  | (0.96, 4.28) |  | (2.11, 10.7) |  | (0.65, 3.24) |  |
| Sedatives Current | 1.05 | 0.836 | 5.81 | **<0.001** | 1.04 | 0.879 | — | — | 3.98 | **<0.001** |
|  | (0.64, 1.67) |  | (3.96, 8.50) |  | (0.62, 1.69) |  | — |  | (2.46, 6.28) |  |
| Hallucinogens Current | 1.24 | 0.558 | 1.83 | 0.071 | 0.93 | 0.858 | 9.13 | **<0.001** | 3.09 | **0.001** |
|  | (0.59, 2.52) |  | (0.93, 3.48) |  | (0.42, 1.96) |  | (4.77, 16.9) |  | (1.54, 5.94) |  |
| Opiods Current | 0.89 | 0.773 | 1.11 | 0.817 | 2.88 | **0.005** | 2.26 | **0.046** | 3.58 | **<0.001** |
|  | (0.42, 1.92) |  | (0.45, 2.69) |  | (1.37, 6.09) |  | (0.98, 4.91) |  | (1.75, 7.15) |  |
| Amphetamines/Khat Current | 3.2 | **<0.001** | 1.89 | **<0.001** | 2.11 | **<0.001** | 3.77 | **<0.001** | — | — |
|  | (2.23, 4.58) |  | (1.33, 2.66) |  | (1.45, 3.04) |  | (2.33, 5.95) |  | — |  |

Note: ^┼┼^ Results from a multivariate logistic regression model; A.O.R = Adjusted Odds Ratio; C.I = Confidence Interval

**Table 5: Independent psychiatric disorders that predict current substance use adjusting for alcohol and substance dependence** ^┼┼^

| **PDSQ Disorders** | **Tobacco Current** | | **Alcohol Current** | | **Cannabis Current** | | **Sedatives Current** | | **Amphetamines/Khat**  **Current** | |
| --- | --- | --- | --- | --- | --- | --- | --- | --- | --- | --- |
|  | A.O.R | Sig. | A.O.R | Sig. | A.O.R | Sig. | A.O.R | Sig. | A.O.R | Sig. |
|  | (95% C.I) |  | (95% C.I) |  | (95% C.I) |  | (95% C.I) |  | (95% C.I) |  |
| Major Depressive Disorder | 1.47 | **0.002** | 1.11 | 0.206 | 1.43 | **0.009** | 1.29 | 0.213 | 0.93 | 0.670 |
|  | (1.14-1.88) |  | (0.95-1.29) |  | (1.09-1.86) |  | (0.86-1.94) |  | (0.66-1.30) |  |
| PTSD | 0.96 | 0.730 | 1.04 | 0.604 | 1.02 | 0.839 | 1.41 | 0.064 | 1.16 | 0.327 |
|  | (0.77-1.20) |  | (0.91-1.19) |  | (0.81-1.30) |  | (0.98-2.02) |  | (0.86-1.54) |  |
| Bulimia/Binge Eating Disorder | 1.14 | 0.537 | 0.95 | 0.716 | 1.29 | 0.275 | 1.21 | 0.540 | 1.36 | 0.248 |
|  | (0.74-1.70) |  | (0.70-1.26) |  | (0.80-1.98) |  | (0.63-2.17) |  | (0.78-2.24) |  |
| OCD | 0.97 | 0.771 | 1.00 | 0.965 | 1.06 | 0.639 | 1.04 | 0.852 | 1.10 | 0.537 |
|  | (0.77-1.22) |  | (0.88-1.14) |  | (0.84-1.34) |  | (0.69-1.60) |  | (0.81-1.50) |  |
| Panic Disorder | 0.96 | 0.772 | 0.89 | 0.146 | 0.85 | 0.23 | 1.04 | 0.836 | 1.36 | 0.059 |
|  | (0.75-1.23) |  | (0.76-1.04) |  | (0.64-1.11) |  | (0.70-1.54) |  | (0.99-1.86) |  |
| Psychosis | 1.32 | **0.016** | 0.89 | 0.069 | 1.07 | 0.549 | 1.13 | 0.538 | 0.86 | 0.316 |
|  | (1.05-1.65) |  | (0.78-1.01) |  | (0.85-1.35) |  | (0.77-1.66) |  | (0.64-1.15) |  |
| Agoraphobia | 0.92 | 0.463 | 0.87 | **0.034** | 0.70 | **0.004** | 0.86 | 0.443 | 0.75 | 0.055 |
|  | (0.74-1.15) |  | (0.76-0.99) |  | (0.55-0.89) |  | (0.60-1.25) |  | (0.56-1.01) |  |
| Social Phobia | 1.06 | 0.615 | 1.40 | **<0.001** | 1.27 | **0.045** | 1.63 | **0.017** | 1.42 | **0.021** |
|  | (0.85-1.32) |  | (1.23-1.59) |  | (1.01-1.59) |  | (1.10-2.45) |  | (1.06-1.90) |  |
| Generalized Anxiety Disorder | 1.06 | 0.666 | 1.40 | **<0.001** | 1.02 | 0.868 | 1.28 | 0.224 | 1.19 | 0.303 |
|  | (0.82-1.36) |  | (1.20-1.64) |  | (0.77-1.35) |  | (0.86-1.88) |  | (0.85-1.64) |  |
| Somatization Disorder | 1.07 | 0.564 | 0.99 | 0.888 | 1.11 | 0.372 | 1.59 | **0.011** | 1.09 | 0.538 |
|  | (0.86-1.33) |  | (0.86-1.13) |  | (0.88-1.40) |  | (1.11-2.27) |  | (0.82-1.46) |  |
| Hypochondriasis | 1.29 | **0.023** | 1.10 | 0.169 | 1.25 | 0.069 | 0.87 | 0.448 | 1.46 | **0.011** |
|  | (1.03-1.62) |  | (0.96-1.26) |  | (0.98-1.58) |  | (0.59-1.26) |  | (1.09-1.96) |  |
| Suicidality | 1.26 | **0.047** | 1.21 | **0.008** | 1.17 | 0.203 | 1.43 | 0.061 | 1.18 | 0.289 |
|  | (1.00-1.58) |  | (1.05-1.39) |  | (0.92-1.50) |  | (0.98-2.07) |  | (0.87-1.59) |  |

Note: ^┼┼^ Results from a multivariate logistic regression model adjusting for alcohol and substance dependence; A.O.R = Adjusted Odds Ratio; C.I = Confidence Interval
